# Black Tea May Be a Prospective Adjunct for Calcium Supplementation to Prevent Early Menopausal Bone Loss in a Rat Model of Osteoporosis

**DOI:** 10.1155/2013/760586

**Published:** 2013-07-24

**Authors:** Asankur Sekhar Das, Maitrayee Banerjee, Dolan Das, Sandip Mukherjee, Chandan Mitra

**Affiliations:** ^1^Pre-Clinical Physiology Laboratory, Tripura Institute of Paramedical Sciences, Hapania, Amtali, Tripura 799 130, India; ^2^Formerly Department of Physiology, Presidency College, Kolkata, West Bengal, India; ^3^Department of Physiology, Hooghly Mohsin College, Chinsurah, Hooghly, West Bengal, India; ^4^Department of Physiology, Kalyani Mahavidyalaya, Kalyani, Nadia, West Bengal, India; ^5^Department of Physiology, Serampore College, 9/1 William Carey Road, Serampore, Hooghly, West Bengal, India

## Abstract

The present study was undertaken to find out the ability of black tea extract (BTE) as a suitable alternative of adjunct for calcium supplementation in treating an ovariectomized rat model of early osteoporosis. Female Wistar rats weighing 140–150 g were divided into four groups consisting of six animals in each group: (A) sham-operated control; (B) bilaterally ovariectomized; (C) bilaterally ovariectomized + BTE; (D) bilaterally ovariectomized + 17**β**-estradiol. Results suggest that BTE could promote intestinal absorption of calcium significantly (*P* < 0.01 for duodenum and ileum; and *P* < 0.05 for jejunum). This was found associated with enhanced activities of two relevant intestinal mucosal enzymes alkaline phosphatase (*P* < 0.01 for duodenum, jejunum, and ileum) and Ca^2+^ activated ATPase (*P* < 0.01 for duodenum, jejunum, and ileum). Such BTE-mediated promotion of calcium absorption was coupled with increase in serum estrogen titer (*P* < 0.01) and recovery of all urinary, bone, and serum osteoporotic marker parameters, including bone histological features. Serum parathyroid hormone level, however, was not altered in these animals (*P* > 0.05). A comparative study with 17**β**-estradiol, a well-known adjunct for calcium supplementation, indicated that efficacy of BTE in maintaining skeletal health is close to that of 17**β**-estradiol. This study suggests that simultaneous use of BTE is promising as a prospective candidate for adjunctive therapies for calcium supplementation in the early stage of menopausal bone changes.

## 1. Introduction

Menopause and increasing age are associated with a decrease in calcium absorption that can contribute to the pathogenesis of osteoporosis [1]. Calcium supplements appear to be effective in reducing bone loss in women, but there is a considerable uncertainty about the effectiveness of calcium supplementation in preventing bone loss at the early stage of the menopause [2–4]. Several adjunctive therapies with calcium supplementation were attempted earlier to prevent osteoporosis in early stage of menopause [5, 6]. Of these different adjunctive therapies, HRT, SERMs, and vitamin D were mostly advised [7, 8], but with controversial results [5, 9, 10]. In addition, HRT and SERMs are associated with well-known adverse health effects like breast cancer [11], thromboembolism [9, 12], stroke, and cardiovascular disease early in the course of therapy [13, 14]. Several phytoestrogens were attempted earlier to prevent osteoporotic damages in menopausal condition. Such studies also had controversial results [15–17]. However, black tea (*Camellia sinensis*) has been reported as a medicinal plant with rich flavonoid content and plethora of health-promoting effects including phytoestrogenic efficacy [18–20], but with no reported adverse health effect. Earlier, it was reported that phytoestrogens have affinity for estrogen receptors (ERs), both *α* and *β* [21] with a more preference for ER-*β* [22]. A recent report has further indicated that, in rat intestine, the predominant estrogen receptor population is beta-type [23]. Earlier, it was also reported that changes of bone metabolism in female rats after ovariectomy are similar to the alterations in early postmenopausal women [24]. In addition, hormone therapy is considered as first-line therapy for preventing bone loss and fractures in early postmenopausal women who are symptomatic [9, 25]. Keeping in view the controversial role of the adjuncts in regulating bone loss in early menopausal condition, the present study was undertaken with black tea to examine its role on intestinal absorption of calcium and its association, if any, with preservation of bone mass in the early phase of menopause. Furthermore, to examine its (BTE) efficacy as an adjunct, a comparative study was undertaken with 17*β*-estradiol. 

## 2. Materials and Methods

### 2.1. Animals and Diets

All animal experiments were performed according to the ethical guidelines suggested by the Institutional Animal Ethics Committee (IAEC) and the Committee for the Purpose of Control and Supervision of Experiments on Animals (CPCSEA), Government of India.

Female Wistar rats weighing 140–150 g were used for this study. Rats were housed in an environmentally controlled animal laboratory and maintained on a 12-hour light/dark schedule at 25 ± 2°C throughout the experimental period. They were acclimated for seven days in laboratory environment and fed with a standard laboratory diet containing 67.36% carbohydrate, 22.7% protein, 5.7% fat, 0.4% calcium, 0.3% phosphorus, and 0.195 nmol vitamin D_3_/g of diet [26]. Also they were given free access to deionized drinking water. After acclimation, rats were regularly checked for two consecutive normal reproductive cycles. They were then randomly divided by initial body weight into four groups consisting of six animals in each group: (A) sham-operated control; (B) bilaterally ovariectomized; (C) bilaterally ovariectomized + BTE; (D) bilaterally ovariectomized + 17*β*-estradiol.

As the changes of bone metabolism in ovariectomized rats are similar to the alterations in early postmenopausal women [24], bilateral ovariectomy was performed to simulate the early menopausal condition. Under light ether anaesthesia, bilateral (dorsolateral) ovariectomies were performed in Groups B, C, and D, and animals of Group A were subjected to sham-operation. They were fed with the same diet as described above. After seven days of recovery from surgical convalescence, animals of Group C were treated orally with 2.5% aqueous black tea extract (BTE) at a single dose of 1 mL/100 g of body weight daily for 28 days [27]. Animals of Group D were treated with 17*β*-estradiol intraperitonially at a single dose of 10 *μ*g/kg [26] of body weight. Animals of other groups were given only deionized water and solvent vehicle as placebo. During the period of BTE and 17*β*-estradiol treatment, Group A was pair-fed with experimental Groups B, C, and D so as to overcome the impact of any altered food intake in the experimental groups. 

### 2.2. Preparation of 2.5% Aqueous BTE

The black tea extract (BTE) was prepared from CTC (Curl, Tear, and Crush) BOP (Broken Orange Pickoe) grade black clonal tea. It was processed and supplied by Tocklai Experimental Station, Jorhat, Assam, India, to the Drug Development Division, Indian Institute of Chemical Biology, Jadavpur, Kolkata. We received a generous gift from that institute, and a fresh 2.5% aqueous BTE was prepared everyday following the method of Wei et al. [28]. 

### 2.3. Preparation of Intestinal Loops

After the experimental period was over, the animals of all groups were fasted for 16 hours. The preparation of animals and intestinal loops for the study of calcium transference *in situ* was made by following the method as described elsewhere by Islam et al. [29]. Briefly, the animal was anaesthetized (urethane, 1.7 mg/kg body wt.), the abdomen of each animal was opened through a midline longitudinal incision, the bile duct was ligated and duodenal, and jejunal and ileal segments were located. Two ligatures, one proximal and the other distal, were applied tightly in each loop measuring about 6–8 cm in all duodenal, jejunal, and ileal segments. Loops were so selected that each contained 8–10 vessels, and care was taken so that no major blood vessels were occluded by the ligature.

### 2.4. Measurement of Intestinal Calcium Transference

For the measurement of intestinal calcium transference, 1 mL of prewarmed (37°C) Tris-HCl buffer solution containing 0.2 mmol CaCl_2_ was injected with a 25-gauge needle in each ligated segment. The intestinal loops were placed in their usual position, and the abdomen was closed. After one hour, animals were sacrificed, the preselected loops were removed and the fluid from each loop was collected separately, together with a few washings of the lumen with triple-distilled water. The calcium content of the collected fluid was then estimated according to the method described by Adeniyi et al. [30]. The difference between the amount of Ca^2+^ introduced and the amount left unabsorbed was used to estimate the amount of Ca^2+^ absorbed. The intestinal part constituting the loop was dried at 90°C to attain a constant weight, which was recorded as the weight of the dried loop. Calcium transference was expressed as *μ*mol/g dry weight/hr.

### 2.5. Preparation of Intestinal Mucosal Extract

After sacrificing the animal and opening of the abdomen, the whole of the small intestine was quickly removed. The portion comprised of the duodenum, jejunum, and ileum were separated and chilled in ice. Intestinal mucosa was collected as described by Maenz and Cheeseman [31], and the scrapings were homogenized according to the method of Koyama et al. [32]. 

### 2.6. Estimation of Enzyme Activities from Intestinal Mucosa

The activity of alkaline phosphatase of intestinal mucosa was estimated using p-nitrophenyl phosphate as substrate [33]. The protein content of the homogenate used for the study was determined using the method of Lowry et al. [34]. The activity of the enzyme Ca^2+^ ATPase was also studied from the mucosal extract using the method of Rorive and Kleinzellar [35]. Phosphate liberated during Ca^2+^ ATPase enzyme activity was estimated by the method of Lowry and Lopez [36]. 

### 2.7. Estimation of Serum Estradiol and Parathyroid (PTH) Hormone Level

Serum was obtained by using standard laboratory protocol. Serum estrogen level (pg/mL) was determined by using the ELISA EIAgen Estradiol kit (Adaltis Italia, Italy), and PTH was determined by ELISA technique by using anti-rat PTH monoclonal antibody (BIO TREND, Germany), goat anti-mouse IgG-HRP and TMB/H_2_O_2_ (GENEI, India). All samples were assayed in duplicate. To avoid interassay variation, all samples were run at one time. The intra-assay coefficients of variation were 9.08% in case of estradiol and 9.45% in PTH. 

### 2.8. Urine Collection

Fasting urine was collected for 24 h (9 a.m. to 9 a.m.) according to the standard laboratory procedure as described elsewhere by Chanda et al. [37]. Care was taken so that no urine was lost through evaporation. Total volume of urine was measured. 

### 2.9. Estimation of Urinary Calcium, Phosphate, Creatinine, and Hydroxyproline

Excretion levels of urinary calcium, phosphate, creatinine, and hydroxyproline were measured according to the methods of Adeniyi et al. [30], Lowry and Lopez [36], R. L. Nath and R. K. Nath [38], and Bergman and Loxley [39], respectively. 

### 2.10. Estimation of Serum Tartrate-Resistant Acid Phosphatase (TRAP) and Alkaline Phosphatase (AP) Activity

Serum tartrate-resistant acid phosphatase (TRAP) activity was estimated spectrophotometrically (Double-Beam Spectrophotometer, Shimadzu 160A; Shimadzu Corporation, Kyoto, Japan) by using the kit (LABKIT, Spain). 

Serum alkaline phosphatase was measured by using p-nitrophenyl phosphate as substrate [33]. Alkaline phosphatase activity was measured by the hydrolysis of *p*-NPP (para nitrophenyl phosphate) at pH 10.8 using glycine-NaOH buffer at 37°C. 

### 2.11. Measurement of Bone Density

For bone density determination, the right femur, eighth thoracic rib, eighth thoracic vertebra, and fourth lumbar vertebra were freed of soft tissue and cleaned. Bone density (g/cm^3^ bone volume) was measured as described by Arjmandi et al. [26] by using Archimedes' principle. Briefly, each bone was put in an unstoppered vial filled with deionized water, and the vial was placed under a vacuum for 90 min to ensure that all the trapped air diffused out of the bone. Each bone was removed from the vial, blotted with gauze sponge, weighed and returned to the vial containing deionized water. The bone was reweighed in water, and density was calculated (g/cm^3^ bone volume). 

### 2.12. Estimation of Bone Ash Content of Calcium and Phosphate

To estimate the mineral content of bones, specific bones were removed and cleaned off adhering soft tissue. The whole bone was extracted two times with a 1 : 1 mixture of ethanol and diethyl ether for 48 h and one time with diethyl ether for 24 h. The dehydrated and defatted bones were ashen for 48 h at 600°C and hydrolyzed in 6 N HCl for determination of calcium and phosphate [40]. Calcium and phosphate were estimated according to the method as described, respectively, by Adeniyi et al. [30] and Lowry and Lopez [36].

### 2.13. Mechanical Testing of Femur

After sacrifice of the animal, left femur was carefully cleaned off adhering adhesive muscles, tendons, and other soft tissues. Bone breaking force was measured as described by Shapiro and Heaney [41] using a hand-held force meter (Excel Enterprises, India). Before testing, each femur was microscopically inspected to ensure that specimen was intact. The loading point was determined as the midpoint of the distance between the greater trochanter and the lateral condyle in the femoral midshaft. This point was exactly at the middle of the distance between the two supports apart from each other by 1.0 cm. The force was applied with the plunger (at a constant rate of 1 mm/min) directly to the femoral midshaft until the sample fractured. Femurs were kept moist with saline during testing. The breaking force (N) was recorded. 

### 2.14. Tissue Collection and Processing for Histological Study

The left proximal tibia and third lumbar vertebra were removed, dissected free of soft tissue, and fixed with 4% paraformaldehyde for 16–18 h at 4°C. Then, specimens were washed for 12 h at 5°C in each of the following series of solutions: 0.01 M PBS containing 5% glycerol, 0.01 M PBS containing 10% glycerol, and 0.01 M PBS containing 15% glycerol. The specimens were then decalcified in EDTA-G solution (14.5 g EDTA, 1.25 g NaOH, and 15 mL glycerol, pH 7.3) for 10–14 days. The decalcified tissues were washed sequentially at 5°C for 12 h in (a) 15% sucrose and 15% glycerol in PBS, (b) 20% sucrose and 10% glycerol in PBS, (c) 20% sucrose and 5% glycerol in PBS, (d) 20% sucrose in PBS; 10% sucrose in PBS, (e) 5% sucrose in PBS, and (f) 100% PBS. Then, the tissues were washed with PBS and dehydrated in a graded series of alcohols, followed by clearing in xylene, and finally embedded in paraffin [42]. The specimens were cut into 5-6 *μ*m sections and stained with hematoxylin-eosin. Representative sections were observed, and photomicrography was performed with the help of a bright-field microscope equipped with a digital camera (Carl Zeiss, Germany).

### 2.15. Data

Data were expressed as mean ± SE. The Kruskal-Wallis nonparametric ANOVA test was performed to find whether or not scores of different groups differ significantly. To test intergroup significant difference, Mann-Whitney *U* multiple-comparison test was performed. StatsDirect software (Version 2.6.5) was used for statistical analysis. Differences were considered significant if *P* < 0.05.

## 3. Results

### 3.1. Intestinal Mucosal Transference of Calcium

Intestinal calcium transference profiles of sham-operated control, ovariectomized, and ovariectomized supplemented with BTE are shown in Table 1. Mucosal transference of calcium was reduced significantly in the ovariectomized rats in all segments when compared with the sham-operated control group (*P* < 0.001 for duodenum; *P* < 0.01 for jejunum and ileum). BTE supplementation could significantly recover such alterations in mucosal transference of calcium in ovariectomized rats (*P* < 0.01 for duodenum and ileum; and *P* < 0.05 for jejunum).

### 3.2. Intestinal Mucosal Alkaline Phosphatase Activity

Results of intestinal alkaline phosphatase activity of different groups of rats are shown in Table 1. Table 1 indicates that, compared with sham-operated group, ovariectomized rats showed significant segment-wise reduction in the activity of alkaline phosphatase in all segments of small intestine (*P* < 0.01 for duodenum, jejunum, and ileum). BTE supplementation was found effective in restoring this enzyme activity in all these segments (*P* < 0.01 for duodenum, jejunum, and ileum).

### 3.3. Intestinal Mucosal Calcium ATPase Activity

Results of intestinal mucosal calcium ATPase activity of different segments of small intestine of rats are shown in Table 1. It was observed that bilateral ovariectomy caused significant reductions in the activity of this enzyme (*P* < 0.01 for duodenum, jejunum, and ileum) when compared with sham-operated control group. But, BTE supplementation in ovariectomized animals could significantly increase this enzyme activity (*P* < 0.01 for duodenum, jejunum, and ileum).

### 3.4. Serum Estradiol and PTH Level

Results of serum estradiol titer of sham-operated control rats, ovariectomized rats, and ovariectomized rats supplemented with BTE are shown in Table 2. As expected, assay indicated that, compared with sham-operated control, a significant decrease in estradiol level was seen in ovariectomized animals (*P* < 0.01). This could be recovered well by BTE supplementation (*P* < 0.01). However, serum PTH level did not show significant variation between sham-operated control, ovariectomized, and ovariectomized supplemented with BTE (Table 2). 

### 3.5. Urinary Calcium, Phosphate, Creatinine, and Hydroxyproline Excretion Profiles and Calcium to Creatinine Ratio

The urinary calcium, phosphate, creatinine, and hydroxyproline excretion profiles together with Ca : Cr ratio of sham-operated control group (Group A), bilaterally ovariectomized (Group B), ovariectomized rats supplemented with aqueous BTE (Group C), and ovariectomized rats supplemented with 17*β*-estradiol (Group D) are shown in Table 3. Compared with sham-operated group, animals of ovariectomized group showed a significant increase in all of the urinary parameters studied, namely, calcium, phosphate, creatinine, and hydroxyproline and Ca : Cr ratio (*P* < 0.01). Elevated responses of all these parameters were significantly counter-regulated in both groups of rats receiving either aqueous BTE (Group C) or 17*β*-estradiol (Group D).

### 3.6. Serum Alkaline Phosphatase (AP) and Tartrate-Resistant Acid Phosphatase (TRAP) Activity

The serum alkaline phosphatase activity profiles of rats of sham-operated control, ovariectomized, ovariectomized + BTE, and ovariectomized + estradiol groups are shown in Table 3. Rats of ovariectomized group (Group B) showed a significant increase in serum AP activity when compared with animals of sham-operated control group (*P* < 0.01) (Group A). This increase in AP activity was significantly lowered (*P* < 0.01) in rats receiving either BTE (Group C) or 17*β*-estradiol (Group D). However, compared with BTE, 17*β*-estradiol supplementation was found more effective in reducing the activity of serum AP in ovariectomized rats. Likewise, the significant increase (*P* < 0.01) in TRAP in ovariectomized animals (Group B), compared with control (Group A), could be effectively reduced by either aqueous BTE treatment (Group C) or 17*β*-estradiol (Table 3). 

### 3.7. Bone Density Profile

Animals in the ovariectomized group (Group B) had significantly lower densities of the right femur (*P* < 0.01), eighth thoracic rib (*P* < 0.01), eighth thoracic vertebra (*P* < 0.01), and fourth lumbar vertebra (*P* < 0.01), compared with the sham-operated control group (Group A). Except thoracic vertebra, BTE supplementation, like 17*β*-estradiol, could produce significant increase (*P* < 0.01) in bone density of all bones: right femur (*P* < 0.01), eighth thoracic rib (*P* < 0.01), eighth thoracic vertebra (*P* < 0.05), and fourth lumbar vertebra (*P* < 0.01) (Table 4). 

### 3.8. Bone Calcium and Bone Phosphate Levels

Results of bone calcium and phosphate levels are shown in Table 4. Animals of ovariectomized group (Group B), compared with sham-operated control group (Group A), showed a marked decrease in calcium and phosphate levels of right femur (calcium: *P* < 0.01; phosphate: *P* < 0.01), eighth thoracic rib (calcium: *P* < 0.01; phosphate: *P* < 0.01), eighth thoracic vertebra (calcium: *P* < 0.01; phosphate: *P* < 0.01), and fourth lumbar vertebra (calcium: *P* < 0.01; phosphate: *P* < 0.01). Significant recovery of both mineral contents of these bones was seen when ovariectomized animals were supplemented with either BTE (Group C) or 17*β*-estradiol (Group D).

### 3.9. Femur Breaking Force

Table 4 shows the results of bone strength of sham-operated control (Group A), ovariectomized (Group B), and ovariectomized rats supplemented with either BTE (Group C) or 17*β*-estradiol (Group D). Compared with sham-operated control (Group A), femurs of ovariectomized rats (Group B) required less force (*P* < 0.01) to fracture. However, a significant recovery (*P* < 0.01) of femur breaking force was noticed when ovariectomized rats were supplemented with either BTE (Group C) or 17*β*-estradiol (Group D) alone. Compared with BTE, 17*β*-estradiol supplementation in these ovariectomized rats showed a better recovery of breaking force of femur. 

### 3.10. Histological Analysis of Cortical and Cancellous Bones

**Figure 1:**
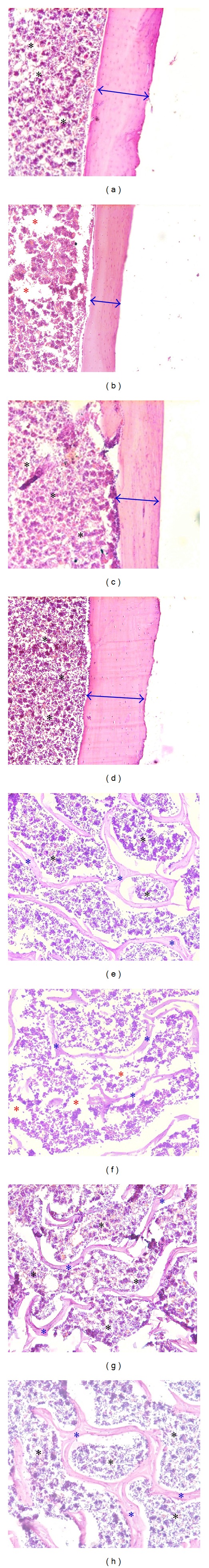
Histological analysis of tibia ((a)–(d)) and lumbar vertebra ((e)–(h)) of rats (hematoxylin-eosin staining, ×100). Representative photomicrograph from ovariectomized group (b) with reduction in cortical thickness and empty bone marrow of the tibia than sham-operated control group (a), ovariectomized + BTE supplemented group (c), and ovariectomized + 17*β*-estradiol-treated group (d). Photomicrograph of lumbar vertebra of ovariectomized group (e) with empty bone marrow and less trabecular bone compared with sham-operated control (d), ovariectomized + BTE supplemented group (g), and ovariectomized + 17*β*-estradiol-treated group (h). Blue line, cortical thickness; black star, bone marrow; red star, empty bone marrow; blue star, trabecular thickness.

Histological study reveals that ovariectomized rats showed a decreased cortical thickness and empty bone marrow at the proximal tibia (Figure 1(b)) as compared to control (Figure 1(a)). Additionally, there was a decrease in trabecular bone volume and thickness at the 3rd lumbar vertebra (Figure 1(f)) compared to control (Figure 1(e)). However, on BTE administration, bone remodeling action was observed in these ovariectomized animals (Figures 1(c) and 1(g)). As expected, similar observation was also found in tibia and lumbar vertebra of the estradiol supplemented group (Group D) (Figures 1(d) and 1(h)). 

## 4. Discussion 

 In the present study, ovariectomized rat was used as an animal model of ovarian hormone deficiency in postmenopausal women [24]. The aim of this study was to examine the prospective role of BTE as an adjunct, because efficacy of several compounds as adjunct with calcium supplementation in the early phase of menopausal bone loss is still uncertain and remains controversial [5, 9, 10]. Additionally, many of these adjuncts are reported to be linked with serious adverse health effects [9, 11–14]. Precisely, this study has demonstrated that supplementation of BTE in an ovariectomized rat model could significantly restore the reduced intestinal absorption of calcium, bone mineral contents, bone histological features, and bone breaking force, with a simultaneous increase in serum estrogen titer, but with no change in PTH level. 

Results of *in vivo* intestinal loop transference studies with the classical technique of Wills [43] indicated that, irrespective of groups, intestinal absorption of Ca^2+^ was reduced in ovariectomized rats. Also, there was a segmental variation in calcium absorption which was in the order of descending gradient from duodenum to ileum. This observation corroborated well with the earlier *in vivo* classical transference studies of Wills [43]. Results of this study furthermore indicated that deficiency of estrogen in ovariectomized rat possibly had a negative influence upon intestinal absorption of calcium, as these animals showed a greater decrease in absorption of calcium than sham-operated control animals (Table 1). Such speculation finds its support from the results of earlier observations with ovariectomized rats [44, 45]. In addition, it has also been reported earlier that estrogen may have direct role via the ERs in regulating intestinal calcium absorption *in vitro* and *in vivo* [46, 47]. In the present study, BTE supplementation was found effective in correcting such reduction in calcium absorption, indicating that BTE possibly has positive influence upon mucosal transference of calcium.

To ascertain the mechanism of such decrease in intestinal absorption of calcium, we examined the activities of the two most relevant mucosal calcium transferring enzymes, namely, alkaline phosphatase and calcium-ATPase. Activities of both of these enzymes were found inhibited in ovariectomized group of animals (Table 1). This agrees well with the earlier observations that both enzymes are linked with calcium absorption as the activities of these enzymes correlate with the degree of calcium absorption in different parts of the intestinal tract under different circumstances [29, 47, 48]. BTE supplementation could well restore the activities of both of these enzymes indicating that the observed positive influence of BTE upon intestinal absorption of calcium was possibly mediated through modulation of the activities of these transferring enzymes. 

To verify whether such ovariectomy-induced alteration in calcium absorption could in any way influence calcium homeostasis and bone functional profiles, a series of parameters were tested. Results revealed that, compared with sham-operated control, ovariectomized rats showed an increase in urinary loss of calcium and phosphate (Table 3). Such an increase in renal excretions of minerals was significantly corrected in ovariectomized animals on receiving BTE, suggesting that BTE has efficacy in retaining bone minerals possibly by preventing bone resorption and/or increasing bone formation or both. Results of studies of the two other specific marker parameters of bone turnover, namely, serum alkaline phosphatase (AP) and urinary calcium to creatinine (Ca : Cr) ratio (Table 3) further support our suggestion. It was observed that BTE was effective in reducing ovariectomy-induced increase in serum AP and urinary Ca : Cr ratio. Since a rise in AP and Ca : Cr ratio is linked with collagen degradation, bone resorption, and osteoporosis [49], it was presumed that this phytoestrogenic compound (BTE) possibly has effective role in reducing bone loss. 

The markers of bone resorption and osteoclastic activity measure circulating or urinary concentrations of fragments of bone matrix that are released during bone resorption, or enzymatic activities associated with osteoclasts. The close association of increased serum concentrations of TRAP and urinary hydroxyproline, respectively, as a potential index for osteoclastic activity and degradation of type I collagen are well established [50]. In the present study, compared with the control, ovariectomized animals showed increased serum TRAP and urinary hydroxyproline (Table 3) levels. These responses were found well regulated by BTE supplementation indicating that BTE possibly is effective also in controlling osteoclastic activity and collagen degradation to prevent skeletal health. Results of histological studies with cortical and cancellous bones (Figures 1(a)–1(d), 1(e)–1(h)) strongly supported this speculation.

Such speculation was further cross-examined in our studies by measuring parameters like bone density and bone mineral content (Table 4). Compared with the control group of animals, bilateral ovariectomy showed low bone densities suggesting that high rate of bone turnover occurred in ovariectomized animals. BTE supplementation could effectively reduce such high rate of bone turnover confirming that BTE has protective action against ovarian hormone deficiency-related bone loss in early menopause. Further confirmatory results were obtained in our experiments with bone mineral content where ovariectomized animals showed lower bone calcium and phosphate content (Table 4), which could be revived significantly after supplementation with BTE. This was further supported by the results of the breaking force of femur (Table 4) because ovariectomy-induced decrease in breaking force of femur was restored by BTE supplementation. 

To assess the efficacy of BTE as a possible agent for adjunct therapy, the results of urinary test parameters of calcium homeostasis and bone functional profiles with BTE were compared with 17*β*-estradiol, the most widely used hormone to prevent early menopausal bone loss. As the results, potency-wise, indicate a close similarity of responses between these two agents (Tables 3 and 4), it may be suggested that BTE has a promise to be an effective adjunct for calcium supplementation in the early phase of menopause, which possibly may be attributed to its phytoestrogenic efficacy in regulating the intestinal absorption of calcium, the mechanism of which may be either by modulating theactivities of mucosal calcium transferring enzymes or by a direct action on intestinal ER or both. Further detailed study on direct intestinal ER activation by BTE is underway.

In summary, BTE has a promise to be an effective adjunct for calcium supplementation in the early phase of menopause. Additionally, it may be used as an anti-osteoporotic naturopathic agent in subjects who are clinically advised not to receive hormone replacement therapy. 

## Figures and Tables

**Table 1:** Intestinal mucosal transference of calcium and calcium-transferring enzymes (alkaline phosphatase activity and calcium-ATPase activity) of different segments of sham-operated control group (Group A), bilaterally ovariectomized group (Group B), and ovariectomized + BTE-treated group (Group C) of rats.

Measures	Sham-operated control (Group A)	Ovariectomized (Group B)	Ovariectomized + BTE (Group C)	Significance level*	Significance level**	
					Group A versus Group B	Group B versus Group C
Calcium transference						
Duodenum	13.25 ± 0.39	9.74 ± 0.27	13.33 ± 0.65	*P* < 0.01	*P* < 0.001	*P* < 0.01
Jejunum	9.69 ± 0.60	6.18 ± 0.45	8.2 ± 0.38	*P* < 0.01	*P* < 0.01	*P* < 0.05
Ileum	5.70 ± 0.28	4.04 ± 0.15	5.67 ± 0.33	*P* < 0.01	*P* < 0.01	*P* < 0.01
Alkaline phosphatase activity						
Duodenum	623.82 ± 9.20	242.62 ± 11.44	525.34 ± 12.71	*P* < 0.001	*P* < 0.01	*P* < 0.01
Jejunum	428.98 ± 4.59	219.70 ± 7.23	356.08 ± 9.41	*P* < 0.001	*P* < 0.01	*P* < 0.01
Ileum	254.78 ± 6.34	141.74 ± 4.86	253.72 ± 5.13	*P* < 0.01	*P* < 0.01	*P* < 0.01
Ca^2+^-ATPase activity						
Duodenum	11.25 ± 0.68	5.61 ± 0.25	10.43 ± 0.52	*P* < 0.01	*P* < 0.01	*P* < 0.01
Jejunum	7.98 ± 0.33	3.71 ± 0.27	8.08 ± 0.20	*P* < 0.01	*P* < 0.01	*P* < 0.01
Ileum	7.01 ± 0.63	2.61 ± 0.16	6.75 ± 0.39	*P* < 0.01	*P* < 0.01	*P* < 0.01

Values are expressed as mean ± SE (*n* = 6); calcium transference is expressed as *μ*mol/g dry weight/hr. Alkaline phosphatase activity is expressed as p-nitrophenol liberated in *μ*mol/g of protein/min at 37°C. Calcium-ATPase activity is expressed as *μ*mol of phosphate liberated/mg of protein/hour at 37°C. *denotes significance level based on the Kruskal-Wallis nonparametric ANOVA test, and **denotes significance level based on Mann-Whitney *U* multiple-comparison test.

**Table 2:** Serum estradiol and PTH levels of sham-operated control group (Group A), bilaterally ovariectomized group (Group B), and ovariectomized + BTE-treated group (Group C) of rats.

Serum levels of hormones	Sham-operated control (Group A)	Ovariectomized (Group B)	Ovariectomized + BTE (Group C)	Significance level*	Significance level**	
					Group A versus Group B	Group B versus Group C
Estradiol (pg/mL of serum)	70.95 ± 1.84	14.81 ± 1.70	41.52 ± 2.44	*P* < 0.001	*P* < 0.01	*P* < 0.01
PTH (pg/mL of serum)	288.78 ± 16.31	276.49 ± 10.16	279.50 ± 9.60	NS	NS	NS

Values are expressed as mean ± SE (*n* = 6); *denotes significance level based on the Kruskal-Wallis nonparametric ANOVA test, and **denotes significance level based on Mann-Whitney *U* multiple-comparison test. NS denotes not significant.

**Table 3:** Urinary excretion of calcium, phosphate, creatinine, and hydroxyproline; calcium : creatinine (Ca : Cr) ratio; and serum alkaline phosphatase (AP) and tartrate-resistance acid phosphatase (TRAP) activities in the sham-operated control group (Group A), bilaterally ovariectomized group (Group B), ovariectomized + BTE-supplemented group (Group C), and ovariectomized + estradiol-treated group (Group D) of rats.

Parameters	Sham-control (Group A)	Ovariectomized (Group B)	Ovariectomized + BTE (Group C)	Ovariectomized + estradiol (Group D)	Significance level*	Significance level**			
						Gr. A versus Gr. B	Gr. B versus Gr. C	Gr. B versus Gr. D	Gr. C versus Gr. D
Urinary parameters									
Calcium (mg/24 h)	0.83 ± 0.06	2.27 ± 0.24	0.90 ± 0.09	0.89 ± 0.06	*P* < 0.01	*P* < 0.01	*P* < 0.01	*P* < 0.01	NS
Phosphate (mg/24 h)	2.74 ± 0.4	4.99 ± 0.18	2.00 ± 0.14	1.76 ± 0.32	*P* < 0.01	*P* < 0.01	*P* < 0.01	*P* < 0.01	NS
Hydroxyproline (mg/24 h)	0.31 ± 0.04	0.69 ± 0.07	0.36 ± 0.05	0.30 ± 0.03	*P* < 0.01	*P* < 0.01	*P* < 0.01	*P* < 0.01	NS
Creatinine (mg/24 h)	1.08 ± 0.12	2.10 ± 0.07	1.00 ± 0.08	1.21 ± 0.14	*P* < 0.01	*P* < 0.01	*P* < 0.01	*P* < 0.01	NS
Ca : Cr	0.73 ± 0.05	1.14 ± 0.08	0.84 ± 0.09	0.77 ± 0.08	*P* < 0.05	*P* < 0.01	*P* < 0.05	*P* < 0.05	NS
Serum parameters									
AP activity (U/L)	151.07 ± 5.58	196.03 ± 8.3	156.70 ± 1.6	100.28 ± 8.45	*P* < 0.001	*P* < 0.01	*P* < 0.01	*P* < 0.01	*P* < 0.01
TRAP activity (U/L)	0.825 ± 0.061	1.725 ± 0.082	0.975 ± 0.122	0.792 ± 0.077	*P* < 0.01	*P* < 0.01	*P* < 0.01	*P* < 0.01	NS

Values are expressed as mean ± SE (*n* = 6). *denotes significance level based on the Kruskal-Wallis nonparametric ANOVA test and **denotes significance level based on the Mann-Whitney *U* multiple-comparison test. NS denotes not significant.

**Table 4:** Body weight, bone density, bone ash content of minerals (calcium and phosphate), and bone breaking force of the sham-operated control group (Group A), bilaterally ovariectomized group (Group B), ovariectomized + BTE-supplemented group (Group C), and ovariectomized + estradiol-treated group (Group D) of rats.

	Sham-control (Group A)	Ovariectomized (Group B)	Ovariectomized + BTE (Group C)	Ovariectomized + estradiol (Group D)	Significance level*	Significance level**			
						Gr. A versus Gr. B	Gr. B versus Gr. C	Gr. B versus Gr. D	Gr. C versus Gr. D
Initial body weight (g)	145.67 ± 3.88	146.33 ± 3.72	145.17 ± 2.92	145.33 ± 3.08	NS	NS	NS	NS	NS
Final body weight (g)	149.83 ± 3.54	179.17 ± 6.34	151.17 ± 2.40	147.33 ± 3.72	*P* < 0.01	*P* < 0.01	*P* < 0.01	*P* < 0.01	NS
Bone density (g/cm^3^)									
Femur	1.3 ± 0.02	1.2 ± 0.02	1.33 ± 0.02	1.38 ± 0.05	*P* < 0.01	*P* < 0.01	*P* < 0.01	*P* < 0.01	NS
Thoracic rib	1.6 ± 0.04	1.33 ± 0.02	1.59 ± 0.04	1.61 ± 0.07	*P* < 0.01	*P* < 0.01	*P* < 0.01	*P* < 0.01	NS
Thoracic vertebra	1.34 ± 0.04	1.17 ± 0.05	1.3 ± 0.01	1.41 ± 0.03	*P* < 0.01	*P* < 0.01	*P* < 0.05	*P* < 0.01	*P* < 0.01
Lumbar vertebra	1.26 ± 0.01	1.16 ± 0.02	1.27 ± 0.02	1.33 ± 0.03	*P* < 0.01	*P* < 0.01	*P* < 0.01	*P* < 0.01	NS
Bone calcium (% of ash weight)									
Femur	21.98 ± 0.64	13.81 ± 0.97	19.95 ± 0.76	20.43 ± 1.24	*P* < 0.01	*P* < 0.01	*P* < 0.01	*P* < 0.01	NS
Thoracic rib	35.49 ± 1.57	24.86 ± 0.31	31.60 ± 1.32	33.49 ± 1.04	*P* < 0.01	*P* < 0.01	*P* < 0.01	*P* < 0.01	NS
Thoracic vertebra	22.95 ± 0.27	11.95 ± 0.64	21.11 ± 0.34	32.76 ± 0.69	*P* < 0.001	*P* < 0.01	*P* < 0.01	*P* < 0.01	*P* < 0.01
Lumbar vertebra	27.01 ± 1.26	18.37 ± 0.37	26.57 ± 1.36	30.83 ± 0.92	*P* < 0.001	*P* < 0.01	*P* < 0.01	*P* < 0.01	*P* < 0.05
Bone phosphate (% of ash weight)									
Femur	19.57 ± 0.28	13.29 ± 0.81	16.14 ± 0.28	16.30 ± 0.19	*P* < 0.001	*P* < 0.01	*P* < 0.01	*P* < 0.01	NS
Thoracic rib	22.03 ± 0.39	18.56 ± 0.49	21.67 ± 0.19	22.36 ± 0.28	*P* < 0.01	*P* < 0.01	*P* < 0.01	*P* < 0.01	NS
Thoracic vertebra	22.02 ± 0.62	17.22 ± 0.51	21.37 ± 0.25	22.61 ± 0.30	*P* < 0.01	*P* < 0.01	*P* < 0.01	*P* < 0.01	NS
Lumbar vertebra	21.02 ± 0.48	16.21 ± 0.20	21.34 ± 0.75	22.16 ± 0.27	*P* < 0.01	*P* < 0.01	*P* < 0.01	*P* < 0.01	NS
Bone breaking force (N)	54.76 ± 2.95	36.78 ± 2.76	49.85 ± 1.51	59.33 ± 2.72	*P* < 0.01	*P* < 0.01	*P* < 0.01	*P* < 0.01	*P* < 0.05

All values are expressed as mean ± SE except for body weight (mean ± SD) (*n* = 6). *denotes significance level based on the Kruskal-Wallis nonparametric ANOVA test and **denotes significance level based on the Mann-Whitney *U* multiple-comparison test. NS denotes not significant.
